# An injector testbed based on a direct current gun and an interchangeable very high frequency gun for superconducting continuous-wave free-electron lasers

**DOI:** 10.1107/S160057752500311X

**Published:** 2025-05-13

**Authors:** Xinmeng Li, Jitao Sun, Yong Yu, Jiahang Shao, Jiayue Yang, Quan Zhou, Hongli Ding, Lei Shi, Kai Tao, Chenglin Guo, Zhigang He, Zhichao Chen, Zhen Chen, Shaohua Peng, Hongfei Wang, Guoqing Zhang, Baichao Zhang, Zongbin Li, Feng Zhao, Wei Wei, Maomao Huang, Wei Wang, Ming Liu, Chaofeng He, Liangbing Hu, Yaqiong Wang, Han Li, Weiming Yue, Xilong Wang, Guorong Wu, Dongxu Dai, Weiqing Zhang, Xueming Yang

**Affiliations:** a Dalian Coherent Light Source and State Key Laboratory of Chemical Reaction Dynamics, Dalian Institute of Chemical Physics Chinese Academy of Sciences Dalian116023 People’s Republic of China; b University of Chinese Academy of Sciences Beijing100049 People’s Republic of China; c Institute of Advanced Light Source Facilities, Shenzhen, Shenzhen518107, People’s Republic of China; d Center for Advanced Light Source, College of Science, Southern University of Science and Technology, Shenzhen518055, People’s Republic of China; SLAC National Accelerator Laboratory, USA

**Keywords:** superconducting radiofrequency, free-electron laser, electron injector, direct current gun, very high frequency gun

## Abstract

The continuous-wave free-electron laser (CW-FEL), based on superconducting radiofrequency (SRF) technology with an electron bunch repetition rate of up to MHz levels, is one of the most advanced light sources, providing exceptionally high average and peak-brightness FEL pulses. Among the new CW-FEL facilities worldwide, the recently proposed Dalian Advanced Light Source (DALS) occupies a unique position as an extreme ultraviolet (EUV) facility primarily designed for chemical physics research. Since the beam emittance requirement for DALS is not as stringent as that for X-ray CW-FEL facilities, a direct current (DC) gun is considered as the primary electron source, with a very high frequency (VHF) gun also planned. To demonstrate key technologies and characterize the electron beam performance, a superconducting CW injector testbed, named the Electron Source Test Facility (ESTF), has been designed and is currently under construction. The testbed is uniquely designed to accommodate both guns with minimal switching effort, where the rest of the beamline layout remains unchanged except for the swapped guns. The two-gun switching scheme for the testbed is shown to be a feasible and cost-effective approach. Furthermore, the injector performance with both guns has been evaluated through a start-to-end simulation based on the DALS configuration, including the production of electron beam in the ESTF injector, the following beam acceleration and compression in a superconducting linear accelerator, and finally the beam lasing performance in the undulator section. The evaluation confirms that the DC gun is a promising electron source for CW-FEL facilities, especially for EUV applications, even though all currently constructed CW facilities have employed the VHF gun. This paper provides a comprehensive description of the injector design and the corresponding performance evaluation.

## Abstract 2

An injector testbed for continuous-wave free-electron lasers based on a direct current gun and an interchangeable very high frequency gun is under construction. Its physical design and performance have been studied carefully.

## 1. Introduction

Free-electron laser (FEL) facilities that can provide high peak brightness FEL pulses have enabled groundbreaking research in both fundamental and applied science. In the past 20 years, several high-gain FEL user facilities based on normal-conducting radiofrequency (NCRF) technology have been constructed and operated worldwide (Emma *et al.*, 2010 ▸; Allaria *et al.*, 2012 ▸; Ishikawa *et al.*, 2012 ▸; Kang *et al.*, 2017 ▸; Yu *et al.*, 2019 ▸; Prat *et al.*, 2020 ▸; Liu *et al.*, 2022 ▸). To meet the growing demands of the scientific community, such as average brightness improvement and multi-user operation capabilities, superconducting radiofrequency (SRF) technology has been adopted in a few FEL facilities, including FLASH (Ackermann *et al.*, 2007 ▸), European XFEL (Decking *et al.*, 2020 ▸), LCLS-II (Brachmann, 2024 ▸), SHINE (Zhao *et al.*, 2024 ▸) and S^3^FEL (Wang *et al.*, 2023 ▸). Among these, FLASH and European XFEL have been successfully commissioned and are currently operating in burst mode with maximum lasing pulse counts of 5000 and 27000 per second, respectively. LCLS-II, SHINE and S^3^FEL are designed to operate in continuous microwave mode with FEL pulse repetition rates of up to about 1 MHz. LCLS-II achieved its formal ‘Threshold Key Performance Parameters’ in 2023 and has been delivering X-ray pulses to experimental areas for first experiments. For SHINE, device installation and initial electron beam commissioning in the accelerator tunnel are underway. Since ground breaking was started in September of 2023, S^3^FEL is currently under construction.

Dalian Coherent Light Source (DCLS) is the first high-gain FEL user facility in China, achieving first lasing in 2016 (Wang, 2017 ▸). It provides FEL pulses in the wavelength range 50–150 nm with a maximum repetition rate of 50 Hz, constrained by the use of NCRF technology (Sun *et al.*, 2024 ▸; Wang *et al.*, 2024 ▸). After several years’ operation with many successful and unique user experiments at DCLS (Li *et al.*, 2024 ▸; Zhou *et al.*, 2023 ▸; Zhang *et al.*, 2020 ▸; Chang, Fu *et al.*, 2023 ▸), a brand-new facility DALS (Dalian Advanced Light Source), based on superconducting technology, has been proposed with the goals of boosting the pulse repetition rate up to 1 MHz and extending the FEL wavelength range to the extreme ultraviolet (EUV) region (Yang, 2021 ▸). The key parameters of DALS are summarized in Table 1 ▸. Compared with synchrotron light sources, DCLS, as an NCRF-based FEL facility, can produce light pulses with peak brilliance several orders of magnitude higher (Barletta *et al.*, 2006 ▸). However, its repetition rate is lower by several orders of magnitude. As an improvement, DALS will combine the high peak brilliance of NCRF FELs with the high repetition rate of synchrotron light sources, significantly advancing scientific progress. Given the photodissociation and photoionization characteristics of EUV light, DALS with MHz-rate and highly bright pulses will serve as an unparalleled cutting-edge tool in atomic and molecular science, astrochemistry, molecular biology, atmospheric chemistry, combustion science and related fields (Ng, 2002 ▸; Chang, Ashfold *et al.*, 2023 ▸; Schlathölter *et al.*, 2016 ▸; Zhang *et al.*, 2024 ▸; Savee *et al.*, 2015 ▸).

A superconducting CW injector is critical to provide a high-quality electron beam (Schmerge *et al.*, 2014 ▸; Wang *et al.*, 2019 ▸; Jia *et al.*, 2024 ▸). The ESTF project containing a fully functional and fully scaled injector has therefore been planned to demonstrate the key technologies and beam quality for DALS. The emittance requirement of DALS is less stringent than that of hard X-ray facilities (1.0 versus 0.4 mm mrad) (Papadopoulos *et al.*, 2014 ▸; Zhu *et al.*, 2017 ▸), which opens up the option of alternative CW electron sources. Compared with very high frequency (VHF) guns chosen by LCLS-II and SHINE, DC guns with lower cathode surface field and voltage offer the following advantages that could benefit facility operation and future upgrade to even higher average brightness. First, although for both VHF and DC guns the photocathode drive laser needs to be synchronized to the RF of the accelerator, DC guns themselves do not require synchronization systems and low-level RF systems for their direct-current voltage operation, thereby simplifying operation and reducing costs. Second, the lower cathode surface field can result in lower dark current, thereby reducing the burden on downstream SRF cavities. Third, the absence of RF heating in DC guns can help to maintain a better vacuum environment that extends the lifetime of semiconductor photocathodes, particularly for the multi-alkali ones with high quantum efficiency illuminated by visible lasers (Seimiya *et al.*, 2014 ▸). Fourth, DC guns can produce beams with higher repetition rates, even reaching SRF cavity frequency of 1.3 GHz (Gulliford *et al.*, 2013 ▸; Akemoto *et al.*, 2018 ▸; Dunham *et al.*, 2007 ▸). Therefore, a 300 kV Cornell-type DC gun (Smolenski *et al.*, 2009 ▸) is taken as the primary source to produce electron beams in the ESTF injector. On the other hand, VHF guns with lower beam emittance could offer a greater margin for engineering considerations. Therefore, a 750 kV APEX-type VHF gun (Zheng *et al.*, 2019 ▸; Zheng *et al.*, 2023 ▸) will also be employed and tested in the injector. These two guns are designed to be interchangeable with minimal modifications to the injector beamline, *i.e.* they share an identical beamline layout downstream of the guns.

In this research, systematic beam dynamics optimization of the ESTF injector has been conducted and the superconducting linear accelerator (LINAC) design as well as the FEL lasing simulation for DALS have been further performed to validate the injector design. This paper is organized as follows: Section 2 focuses on the beam dynamics optimization of the injector, Sections 3 and 4 present the LINAC design and FEL lasing simulation to verify the injector performance, and Section 5 concludes the study.

## 2. Injector optimization

**Figure 1:**
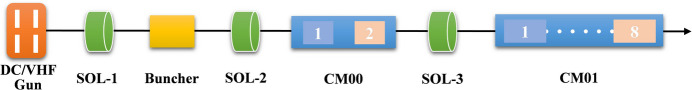
Physical layout of the ESTF injector.

The major beamline components of the ESTF injector are arranged as shown in Fig. 1 ▸. Compared with S-band guns widely used in low repetition rate facilities (Zheng *et al.*, 2016 ▸), DC and VHF guns operate with significantly lower cathode surface field (<30 MV m^−1^ versus 100 MV m^−1^) and lower accelerating voltages. Consequently, the temporal pulse length of the drive laser needs to be of the order of tens of pico­seconds to mitigate emittance growth from the space charge effect, and a 340 kV CW NCRF buncher following the gun is employed to impose a negative energy chirp for velocity bunching (Gao, Zheng *et al.*, 2024 ▸). Finally, the beam energy is boosted to about 100 MeV in a cryomodule containing eight nine-cell TESLA-type SRF cavities (CM01) (Hou *et al.*, 2019 ▸). A short cryomodule containing two two-cell SRF cavities (CM00) is positioned between the buncher and CM01 to pre-accelerate the electron beam and consequently help to improve the beam quality (Gulliford *et al.*, 2013 ▸; Gulliford *et al.*, 2015 ▸; Akemoto *et al.*, 2018 ▸). Additionally, three magnetic solenoids (SOL1-3) are interspersed among the above RF components for space charge effect compensation and electron beam size control. The buncher and the SRF cavities operate at an RF frequency of 1.3 GHz, while the VHF gun operates at 216.67 MHz (one-sixth of 1.3 GHz).

The beam dynamics optimization aims to obtain the Pareto front of the normalized emittance and the bunch length at the injector exit to determine the optimal working point. The optimization was performed by combining the multi-objective genetic algorithm (MOGA) with the *ASTRA* code (Murata & Ishibuchi, 1995 ▸; Flöttmann, 2011 ▸; Jazzbin, 2020 ▸). It includes the strength constraints of RF elements and solenoids as well as the mechanical constraints to ensure sufficient space for non-electromagnetic components (the laser box, bellows, beam instruments, *etc*.). Variables such as the transverse size (1σ truncated Gaussian distribution) and the temporal duration (plateau distribution) of the drive laser, the longitudinal positions of the beamline components, the voltages and phases of all RF cavities, and the field strengths of the solenoids, were considered in the optimization. The electromagnetic fields of the DC/RF cavities and the solenoids were simulated using *CST Microwave Studio* and *Opera Simulation Software* (CST Studio Suite, 2022 ▸; Opera, 2022 ▸), respectively. The thermal emittance was set to 1 mm mrad mm^−1^, which is consistent with the typical measured values of Cs_2_Te cathodes (Filippetto *et al.*, 2015 ▸; Prat *et al.*, 2015 ▸; Zheng *et al.*, 2020 ▸).

The remainder of this section is divided into four parts. First, the injector layout with the DC gun is optimized under the nominal operation conditions (a gun voltage of ∼300 kV and a bunch charge of 100 pC) and the corresponding working point is selected, which fixes the positions of all downstream components. Second, based on the fixed component positions, the injector beam dynamics based on the VHF gun is optimized, and the results based on both guns are compared. Third, the injector performance is explored over a wider range of gun voltages and bunch charges. Finally, the feasibility of switching the two guns with corresponding optimal beamline settings is analyzed, and key technologies are investigated.

### 2.1. DC gun injector

**Figure 2:**
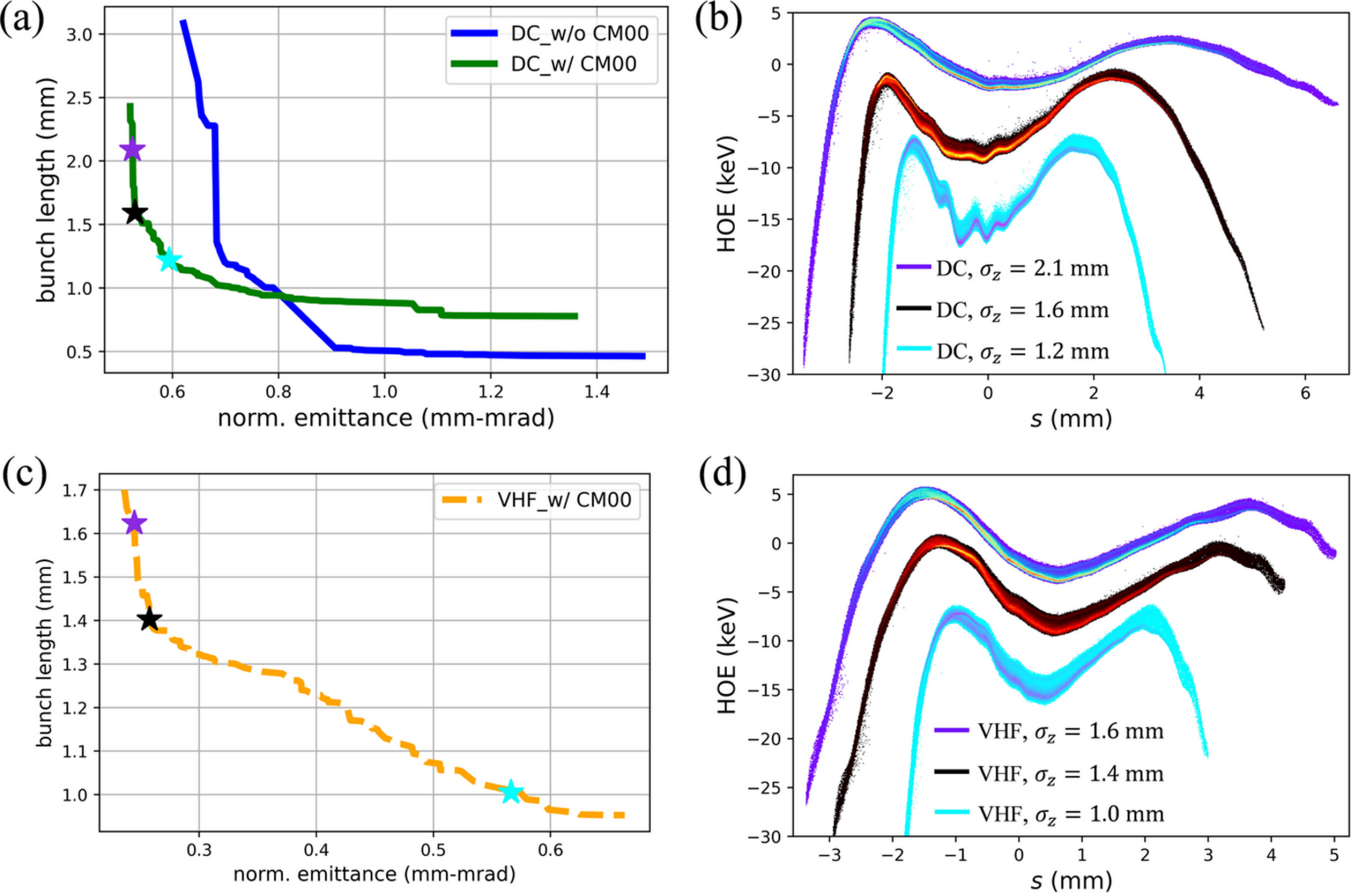
Optimized results of the ESTF injector with ∼300 kV DC gun (*a*, *b*) and 750 kV VHF gun (*c*, *d*). (*a*, *c*) Pareto fronts of the normalized emittance and bunch length; (*b*, *d*) HOE distributions of several typical points on the Pareto fronts with CM00: RMS bunch lengths σ_*z*_ of 1.2/1.6/2.1 (1.0/1.4/1.6) mm and corresponding RMS HOEs of 3.9/3.3/3.2 (4.0/3.9/3.9) keV for the DC (VHF) gun injector.

Two beamline configurations with and without the short cryomodule CM00 were considered, and the optimization results are shown in Fig. 2 ▸(*a*). Although both configurations are capable of meeting the normalized emittance requirement (ɛ_n_ < 1.0 mm mrad), it is preferable to preserve a lower emittance for potential emittance deterioration in a realistic facility. Compared with the configuration without CM00, the configuration with CM00 retains a shorter bunch length at a lower emittance, which is more advantageous for the subsequent bunch compression in an SRF LINAC. Additionally, only the configuration with CM00 can provide an ultimately low emittance (ɛ_n_ < 0.6 mm mrad), which is more beneficial for the FEL lasing. Therefore, the CM00 was included in the injector layout, as shown in Fig. 1 ▸.

Next, the higher-order energy spread (HOE) of the electron beam was investigated, as a lower HOE is preferred for the subsequent bunch compression in a LINAC (Chen *et al.*, 2022 ▸). Fig. 2 ▸(*b*) shows three HOE distributions that correspond to the typical points marked with stars in Fig. 2 ▸(*a*). It indicates that a shorter bunch length leads to more severe microbunching modulation as well as a higher HOE. The nominal working point for the DC gun injector was finally determined at an RMS bunch length of 1.6 mm with a good balance among normalized emittance, bunch length and HOE. The resultant optimized positions and other variables of the beamline components are summarized in Table 2 ▸.

### 2.2. VHF gun injector

**Figure 3:**
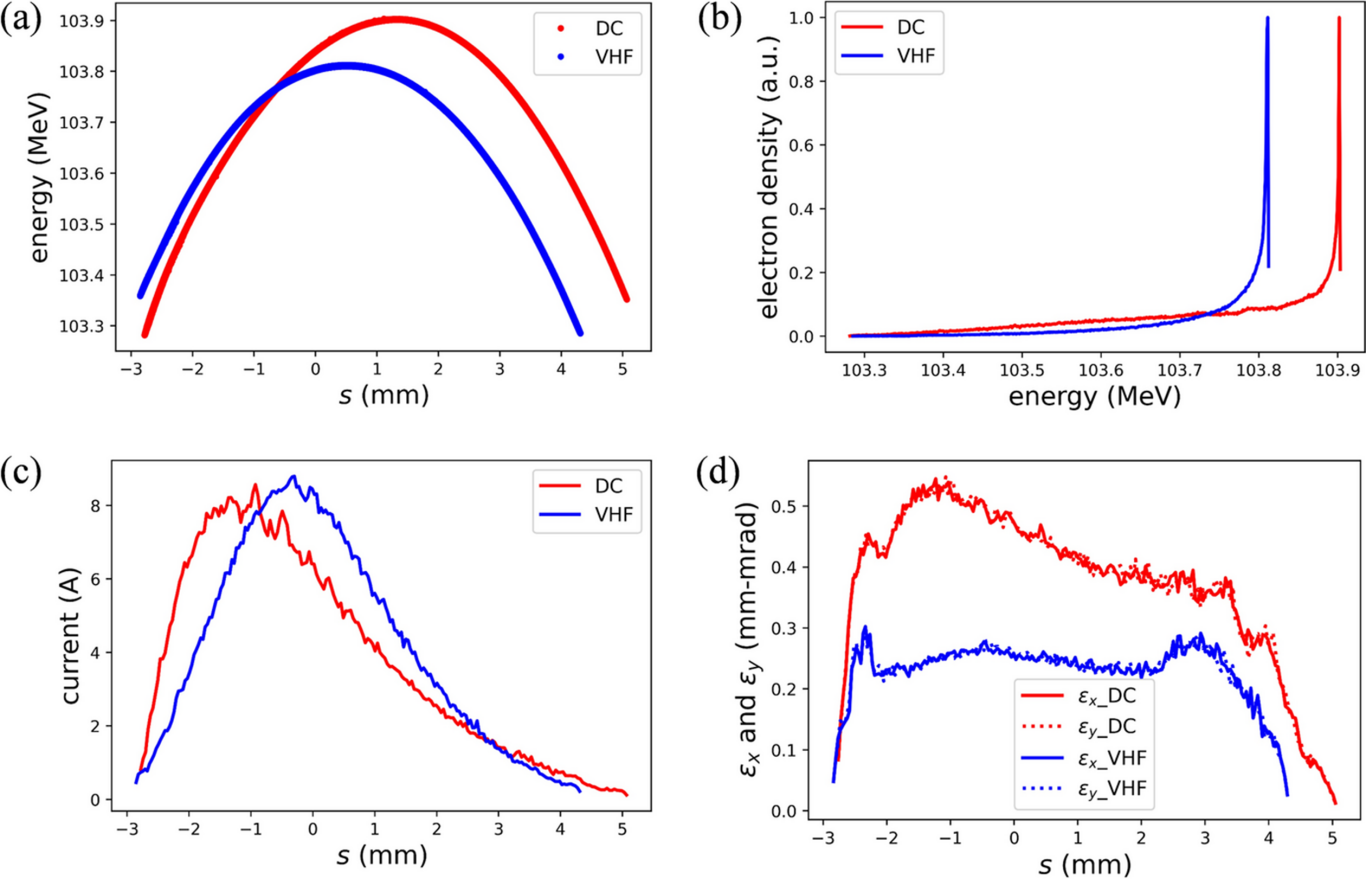
Nominal beam performance at the injector exit for the DC (red) and VHF (blue) gun cases. (*a*) Longitudinal phase spaces, (*b*) energy distributions, (*c*) current distributions, and (*d*) normalized slice emittances in the *x* and *y* directions.

For the VHF gun case, the same beamline components and positions as those in the DC gun case were used, as listed in Table 2 ▸. Using the MOGA method based on *ASTRA*, the optimal Pareto front was obtained, as shown in Fig. 2 ▸(*c*). The corresponding HOE distributions for three typical points on the Pareto front are shown in Fig. 2 ▸(*d*). Following the same criteria as the DC gun case, the nominal working point for the VHF gun injector was determined at an RMS bunch length of 1.4 mm. The optimized variables of the VHF gun case are compared with those of the DC gun case in Table 2 ▸. The resultant beam performance is presented in Fig. 3 ▸. It is obvious that the resulting nominal beam properties are basically similar in both cases, except that the emittance of the VHF gun case is 50% smaller, as also shown in Table 3 ▸. Nevertheless, the emittances in both cases meet the requirements of DALS (refer to Table 1 ▸).

### 2.3. Extended operation range of ESTF injector

**Figure 4:**
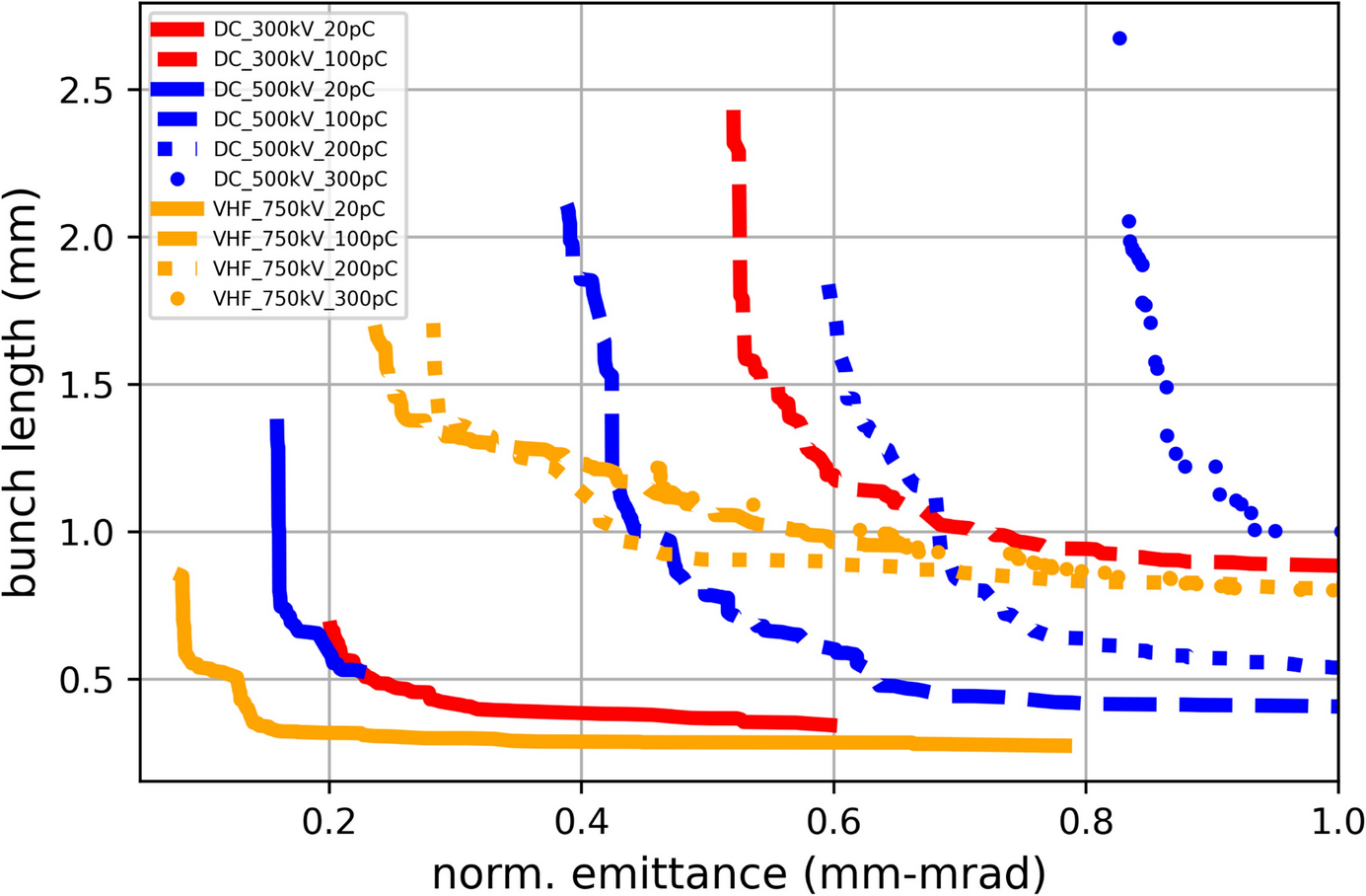
Pareto fronts of the ESTF injector for various operation conditions, labeled by gun types, gun voltages and bunch charges.

In this part, the injector performance has been explored with the gun voltage of 300–750 kV as well as the bunch charge of 20–300 pC. Higher DC gun voltage is desired for improving emittance or shortening bunch length in future upgrades. It can be achieved by using a shorter anode, *i.e.* increasing the cathode–anode distance. The adjustment of bunch charge is motivated by various user experimental requirements and can be conducted by tuning the drive laser energy without any mechanical modification to the beamline components. In the certain calculations, voltages of 300/500 kV for the DC gun and 750 kV for the VHF gun were evaluated, along with the bunch charges of 20/100/200/300 pC. The same MOGA method as mentioned above was used in the evaluation but with the positions of all beamline components fixed (as listed in Table 2 ▸). The resultant Pareto fronts at the injector exit are illustrated in Fig. 4 ▸. As expected, a higher charge deteriorates the beam performance due to the stronger space charge effect while a higher gun voltage improves the performance for the better space charge effect suppression. A summary of the beam performance variations under various gun voltages and bunch charges is given in Table 3 ▸.

### 2.4. Feasibility consideration of switching two guns

**Figure 5:**
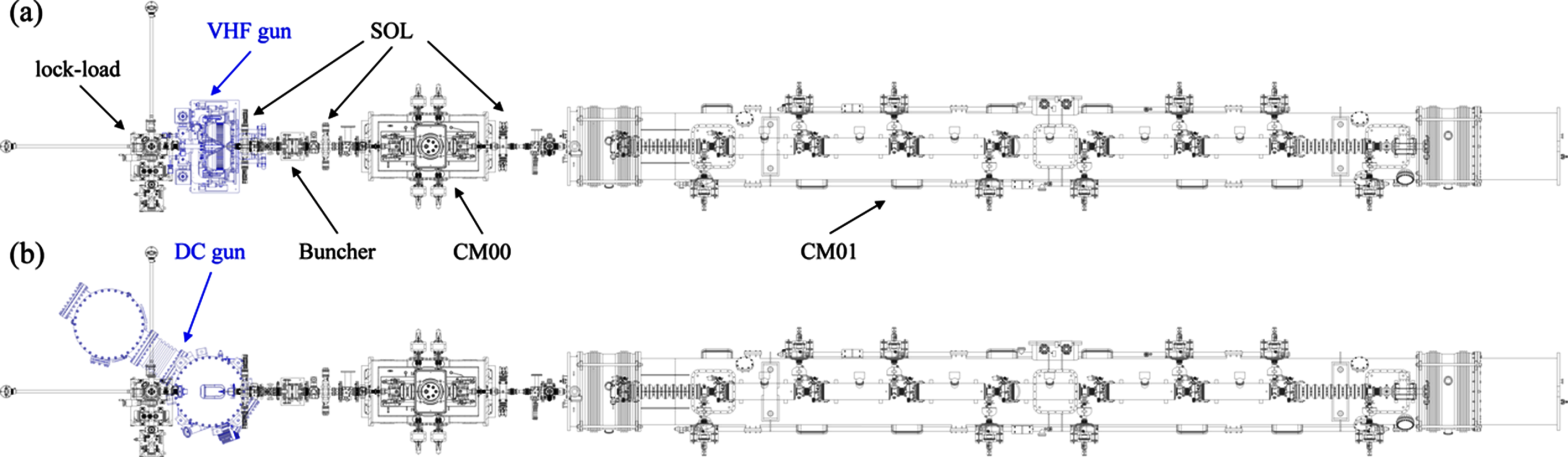
Mechanical layout of the ESTF injector with interchangeable VHF gun (*a*) and DC gun (*b*).

The feasibility of switching the DC gun and the VHF gun in the ESTF injector was considered to support the optimized beamline settings presented in Table 2 ▸. As shown in Fig. 5 ▸, the beamline components, except for the guns, remain unchanged in the mechanical layout. The upstream and downstream flanges of the guns are connected to the cathode lock-load chamber and the vacuum pipe covered by the first solenoid (SOL-1), respectively. It provides a flexible way for the gun switching by disconnecting and reconnecting these flanges. When the gun is switched, the physical parameters of the corresponding components need to be regulated accordingly, as shown in Table 2 ▸. Except for the drive laser and the buncher parameters, the other parameters do not differ significantly between the two-gun cases and can be easily achieved. Consequently, some additional efforts were made for the drive laser and the buncher. First, the laser pulses with plateau distribution were obtained by stacking short pulses generated by a compressor, utilizing both α-BBO crystals and Michelson interferometers (Zhang *et al.*, 2023 ▸). The duration of the resulting stacked pulses can be adjusted by modifying the compressor settings, as well as the configurations of the α-BBO crystals and the Michelson interferometers. The laser transverse diameter with a 1σ truncated Gaussian distribution is achieved by adjusting the original laser size using a telescope and then cutting the size to a ±1σ diameter using an aperture. Therefore, it can be adjusted by modifying the appropriate telescope setting and aperture size. Second, the NCRF buncher is designed with adequate cooling channels to handle the heat load due to RF power. Its resonant frequency can be tuned to 1.3 GHz within the required voltage range by adjusting the cooling water temperature, which induces thermal expansion and contraction (Gao, Zheng *et al.*, 2024 ▸).

Therefore, it can be concluded that the component layout and setups can accommodate both guns with minimal switching effort, which makes ESTF a convenient testbed to evaluate the injector performance for both DC and VHF guns.

## 3. Performance verification of the ESTF injector beam in the DALS LINAC

**Figure 6:**
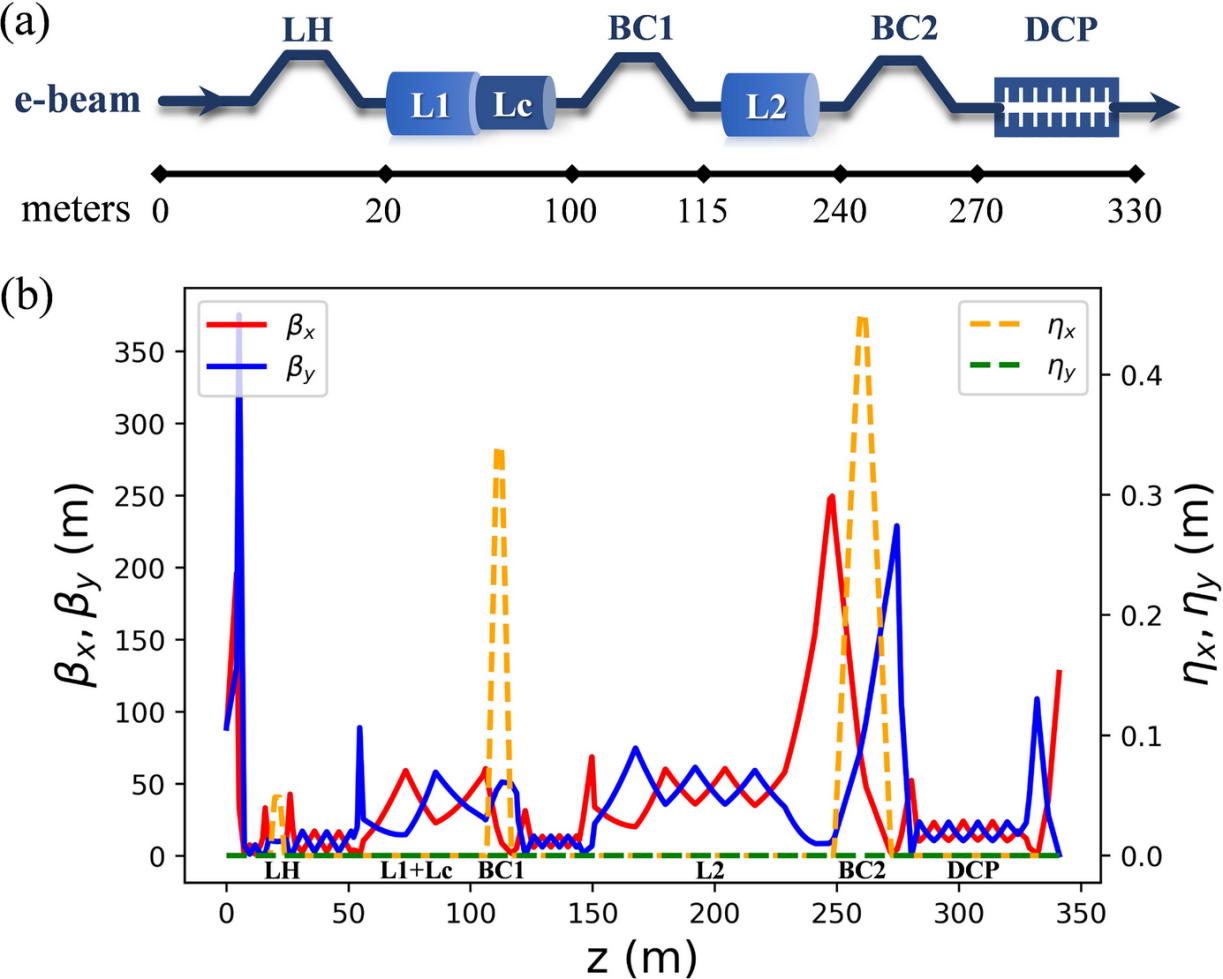
Schematic layout (*a*) and Twiss parameters (*b*) of the DALS LINAC.

To verify the injector performance, the beams produced by the ESTF injector were tracked in the DALS SRF LINAC section and subsequently evaluated for FEL lasing performance in the undulator. The major component layout of the LINAC is shown in Fig. 6 ▸(*a*). Following the injector, a laser heater (LH) is used to suppress the microbunching instability (Huang *et al.*, 2004 ▸). The beam is accelerated in two sections (L1 and L2) with two and seven standard 1.3 GHz cryomodules (Pan *et al.*, 2024 ▸), respectively. Meanwhile, the bunch length is compressed by two-stage magnetic bunch compressors (BC1 and BC2). An RF linearizer (Lc), consisting of two 3.9 GHz cryomodules, is located before BC1 to enhance compression efficiency (Edwards *et al.*, 2010 ▸). A 20 m-long wake field dechirper (DCP), consisting of five pairs of 2 m × 2 m orthogonal planar structures with corrugated plates, is used to compensate the energy chirp imposed by L1 and Lc (Zhang *et al.*, 2015 ▸). The gap between the two plates is 3.0 mm, while other physical parameters of the plates are the same as that used at LCLS (Guetg *et al.*, 2016 ▸). The transverse lattice is designed to minimize the emittance growth, employing several strategies such as the use of FODO cells, appropriate control of phase advances, small beta functions at the last magnetic bend of BC1 and BC2, and the orthogonal arrangement of neighboring dechirper plates (Holzer, 2016 ▸; Zhang *et al.*, 2015 ▸). The evolution of Twiss parameters (beta functions β_*x*_, β_*y*_ and dispersion functions η_*x*_, η_*y*_) (Courant & Snyder, 1958 ▸) along the LINAC beamline is shown in Fig. 6 ▸(*b*).

**Figure 7:**
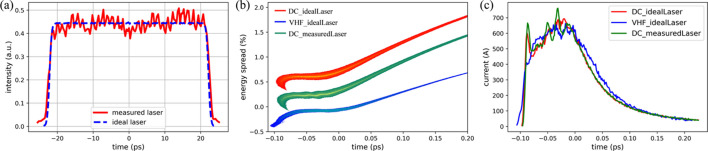
Simulation results for the DALS LINAC. (*a*) The temporal distributions of ideal (blue) and measured (red) drive lasers used to generate electron beams in ESTF injector, (*b*) the beam longitudinal phase spaces, constant energy deviations were added to avoid overlap, and (*c*) current distributions at the LINAC exit for DC gun cases with the ideal/measured laser (red/green) and VHF gun case with ideal laser (blue).

Based on this LINAC design, both the electron beams produced in the DC gun and VHF gun cases were evaluated. The drive lasers with a plateau temporal distribution were adopted to produce the initial electron beam in the injector. For the ideal laser case, related to the nominal parameters listed in Table 3 ▸, the laser with a smooth temporal distribution was generated using *ASTRA* code. However, the actual laser stacked by short pulses in a laboratory usually exhibits a non-smooth temporal distribution that may lead to serious beam microbunching instability (Bettoni *et al.*, 2020 ▸). Therefore, to consider this effect, the actual laser distribution was measured in our laboratory (Gao, Li *et al.*, 2024 ▸) and incorporated into the electron beam dynamics simulations. This measured distribution and the corresponding ideal one are shown in Fig. 7 ▸(*a*), showing distinct microstructures for the former. Both distributions are used in the DC gun case. For the VHF gun case, only the ideal laser distribution was considered due to the higher gun voltage and the consequently weaker space charge effect.

The beam dynamics simulations were performed with the *ELEGANT* code (Borland, 2000 ▸), in which the longitudinal space charge effect, coherent synchrotron radiation effect, SRF cavity wake fields (Novokhatski *et al.*, 1999 ▸; Weiland & Zagorodnov, 2003 ▸; Zagorodnov *et al.*, 2004 ▸) and dechirper wake fields were included. Table 4 ▸ lists the LINAC design parameters and corresponding beam performance for both the DC and VHF gun cases. The detailed beam distributions at the exit of the LINAC including the longitudinal phase spaces and current distributions are shown in Figs. 7 ▸(*b*) and 7 ▸(*c*), respectively. The simulation results indicate that the beam parameters required by DALS, as listed in Table 1 ▸, were achieved for both the DC and VHF gun cases, which validates the ESTF injector design. Typically, the emittance growth in both cases is well controlled and the emittance in the VHF gun case is still approximately 50% lower than that in the DC gun case. Additionally, in the DC gun injector, the LINAC beam results were compared under both ideal and measured laser conditions. The same configurations, including the injector and LINAC, were used for both conditions. Their corresponding LINAC beam parameters are nearly identical, as shown in Table 4 ▸. This indicates the robustness of the physics design. However, the microbunching structures in beam energy and current density modulation are distinct in the measured laser condition, even with the use of a laser heater. The subsequent section will present an evaluation of the FEL lasing performance.

## 4. FEL lasing performance based on ESTF injector beams

Following the LINAC beam results, the FEL lasing performance was further investigated as the best touchstone of the ESTF injector. This investigation focused on the minimal DALS FEL wavelength of 6.5 nm in self-amplified spontaneous emission (SASE) mode (Kondratenko & Saldin, 1980 ▸; Bonifacio *et al.*, 1984 ▸), which demands the tightest beam quality. The FEL lasing simulation in the undulator was conducted using the *GENESIS* code (Reiche, 1999 ▸), employing 16 tapered undulators (Kroll *et al.*, 1981 ▸) with a period of 34 mm, a single segment length of 4 m and an intersegment spacing of 1 m.

**Figure 8:**
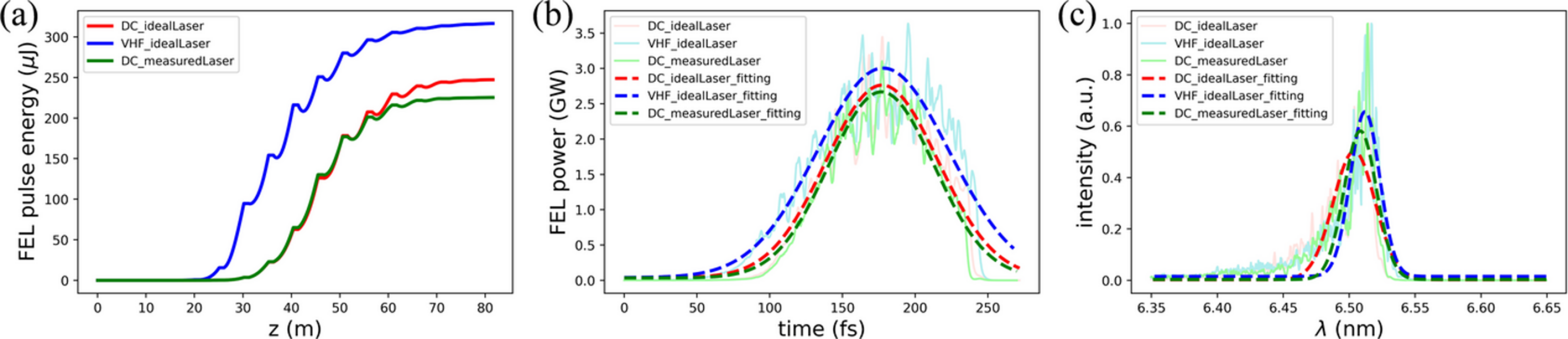
FEL lasing performance based on DC (red, green) and VHF (blue) gun ESTF injectors. (*a*) Gain curves of the FEL pulse energy, (*b*) time-domain FEL power distributions and (*c*) corresponding FEL spectra.

Since SASE radiation starts up from shot-noise microbunching, 20 random shot noises were considered in the simulation to evaluate their averaging effect. These averaging results are presented in Fig. 8 ▸ with key lasing parameters summarized in Table 4 ▸. Due to the larger beam emittance in the DC gun case, the corresponding FEL pulse energy is about 20% lower than that in the VHF gun case. Comparing the FEL lasing performance between the measured and ideal drive laser cases for the DC gun injector, the FEL pulse energy for the measured case is about 10% lower than that for the ideal case. This indicates that the drive laser with a temporal distribution stacked by short pulses deteriorates the FEL lasing performance to some extent. Figs. 8 ▸(*b*) and 8 ▸(*c*) show the calculated FEL power distributions and spectra, along with the corresponding Gaussian fitting ones, where there is no significantly distinct difference in FEL peak power or spectral bandwidth between the DC and VHF gun cases. These results suggest that the ESTF injector can provide promising beams for DALS, and the DC gun can fulfill FEL lasing requirements in the EUV region with performance comparable with that of the VHF gun.

## 5. Conclusion

In this study, we have performed detailed optimization of the ESTF, which is currently under construction as an electron beam injector testbed for the proposed superconducting continuous-wave EUV FEL facility, DALS. The testbed was designed to be compatible with both the DC and VHF guns. The design optimization has accounted for both physical and engineering considerations, achieving a balance among the beam properties of normalized emittance, bunch length and higher-order energy spread. Based on the optimized positions of the beamline components, the injector performance with various gun voltages and bunch charges was systematically studied, providing the operation range of the injector for future reference. The engineering feasibility of the interchangeability of the two guns on the beamline has also been carefully considered. In addition, based on the ESTF injector beams, the DALS 1 GeV SRF LINAC was designed and the corresponding FEL lasing performance at the minimum wavelength of 6.5 nm was evaluated. The results confirm that the ESTF injector with both DC gun and VHF gun can fulfill the requirements of the proposed DALS. The main components of the ESTF beamline are currently being installed according to the injector design and the first beam is expected to be launched in the first half of 2025.

## Figures and Tables

**Table 1:** Key parameters of DALS

Parameter	Value	Units
Electron energy	1	GeV
Bunch charge	100	pC
Peak current	600	A
Normalized emittance, ɛ_n_†	<1.0	mm mrad
Repetition rate	1	MHz
FEL wavelength	6.5–180	nm
FEL pulse energy	>50	µJ

† ɛ_n_ is calculated at the minimum wavelength λ_min_ = 6.5 nm according to the diffraction-limited condition βγλ_min_/4π, where γ and β denote the Lorentz factor and relativistic velocity, respectively.

**Table 2:** Optimal variables of the beamline components for the ESTF injector

Component	Parameter	DC injector	VHF injector	Units
Drive laser	Temporal duration (FWHM)	42.6	32.4	ps
	Transverse diameter	1.51	0.85	mm
Gun	Position in beamline	0	0	m
	Voltage	311	750	kV
	RF phase	–	0	degrees
	Cathode surface field	6.5	26	MV m^−1^
Buncher	Position in beamline	0.997	0.997	m
	Voltage	46.8	208	kV
	RF phase	−75.2	−61.3	degrees
CM00, cavity 1 and 2	Position in beamline	2.668 and 3.277	2.668 and 3.277	m
	Voltage	1.6 and 2.1	2.0 and 2.1	MV
	RF phase	2.5 and −24.8	−20.0 and −8.4	degrees
CM01, cavity 1	Position in beamline	6.424	6.424	m
	Voltage	13.7	10.8	MV
	RF phase	−6.2	−3.1	degrees
CM01, cavity 2–8	Position in beamline (equally spaced)	7.808–16.109	7.808–16.109	m
	Voltage	10.7–16.1	10.7–16.1	MV
	RF phase	0	0	degrees
SOL-1	Position in beamline	0.300	0.300	m
	Focal strength	7.1 × 10^−5^	25.4 × 10^−5^	T^2^ m
SOL-2	Position in beamline	1.475	1.475	m
	Focal strength	3.0 × 10^−5^	6.8 × 10^−5^	T^2^ m
SOL-3	Position in beamline	4.059	4.059	m
	Focal strength	52.1 × 10^−5^	43.6 × 10^−5^	T^2^ m

**Table 3:** Beam performance of the ESTF injector under various operation conditions

Parameter	Nominal	Range	Units
Gun voltage	DC: 311	300–750	kV
	VHF: 750		
Bunch charge	100	20–300	pC
Beam energy	100	–	MeV
Bunch repetition rate	1	0–1	MHz
Normalized emittance	DC: 0.53	0.1–1.0	mm mrad
	VHF: 0.26		
Peak current	DC: 8	2–30	A
	VHF: 9		
RMS bunch length	DC: 1.6	0.4–2.5	mm
	VHF: 1.4		

**Table 4:** Design parameters of the DALS LINAC based on DC and VHF gun injectors

Parameter	DC with ideal laser	DC with measured laser	VHF with ideal laser	Units
LINAC configuration				
L1 voltage	226.6	226.6	224.8	MV
L1 phase	−8.2	−8.2	−12.5	degrees
Lc voltage	62.2	62.2	62.6	MV
Lc phase	−146.6	−146.6	−150.9	degrees
L2 voltage	831.1	831.1	750.0	MV
L2 phase	−24.1	−24.1	−63.3	degrees
BC1 R56	−56.2	−56.2	−58.9	mm
BC2 R56	−37.6	−37.6	−34.1	mm
Energy loss @ DCP	9.4	9.4	7.4	MeV

Electron beam parameters at the exit of LINAC				
Average beam energy	1.023	1.023	1.004	GeV
RMS bunch length	67.2	67.3	65.2	fs
Peak current	∼600	∼600	∼600	A
Normalized emittance (*x*/*y*)	0.56/0.56	0.57/0.56	0.30/0.28	mm mrad

FEL lasing performance				
FEL wavelength	6.51	6.51	6.52	nm
Pulse energy	247.3	225.4	316.6	µJ
Pulse duration (FWHM)	90.9	85.4	106.7	fs
Peak power	2.76	2.67	3.00	GW
Spectrum bandwidth (FWHM)	0.58	0.45	0.41	%
